# Temporal changes in CT-derived fractional flow reserve in patients after heart transplantation

**DOI:** 10.1007/s00330-024-10932-z

**Published:** 2024-07-17

**Authors:** Simran P. Sharma, Javier Sanz, Alexander Hirsch, Richa Patel, Alina A. Constantinescu, Maya Barghash, Donna M. Mancini, Jasper J. Brugts, Kadir Caliskan, Yannick J. H. J. Taverne, Olivier C. Manintveld, Ricardo P. J. Budde

**Affiliations:** 1 Department of Cardiology, Thorax Centre, Erasmus MC, University Medical Centre Rotterdam, Rotterdam, The Netherlands; 2 Department of Radiology and Nuclear Medicine, Erasmus MC, University Medical Center, Rotterdam, The Netherlands; 3 Cardiovascular Institute, Mount Sinai Hospital, New York, NY USA; 4 Department of Internal Medicine, Mount Sinai Hospital, New York, NY USA; 5 Erasmus MC Transplant Institute, Erasmus MC, University Medical Centre Rotterdam, Rotterdam, The Netherlands; 6 Department of Cardiothoracic Surgery, Erasmus MC, University Medical Centre Rotterdam, Rotterdam, The Netherlands

**Keywords:** Computed tomography angiography, Heart transplantation, Fractional flow reserve, Myocardial

## Abstract

**Background:**

Adding functional information by CT-derived fractional flow reserve (FFRct) to coronary CT angiography (CCTA) and assessing its temporal change may provide insight into the natural history and physiopathology of cardiac allograft vasculopathy (CAV) in heart transplantation (HTx) patients. We assessed FFRct changes as well as CAV progression over a 2-year period in HTx patients undergoing serial CT imaging.

**Methods:**

HTx patients from Erasmus MC and Mount Sinai Hospital, who had consecutive CCTAs 2 years apart were evaluated. FFRct analysis was performed for both scans. FFRct values at the most distal point in the left anterior descending (LAD), left circumflex (LCX), and right coronary artery (RCA) were measured after precisely matching the anatomical locations in both analyses. Also, the number of anatomical coronary stenoses of > 30% was scored.

**Results:**

In total, 106 patients (median age 57 [interquartile range 47–67] years, 67% male) at 9 [6–13] years after HTx at the time of the baseline CCTA were included. Median distal FFRct values significantly decreased from baseline to follow-up for the LAD from 0.85 [0.79–0.90] to 0.84 [0.76–0.90] (*p* = 0.001), LCX from 0.92 [0.88–0.96] to 0.91 [0.85–0.95] (*p* = 0.009), and RCA from 0.92 [0.86–0.95] to 0.90 [0.86–0.94] (*p* = 0.004). The number of focal anatomical stenoses of > 30% increased from a median of 1 [0–2] at baseline to 2 [0–3] at follow-up (*p* = 0.009).

**Conclusions:**

The distal coronary FFRct values in post-HTX patients in each of the three major coronary arteries decreased, and the number of focal coronary stenoses increased over a 2-year period. Temporal FFRct change rate may become an additional parameter in the follow-up of HTx patients, but more research is needed to elucidate its role.

**Clinical relevance statement:**

CT-derived fractional flow reserve (FFRct) is important post-heart transplant because of additional information on coronary CT angiography for cardiac allograft vasculopathy (CAV) detection. The decrease and degree of reduction in distal FFRct value may indicate progression in anatomic CAV burden.

**Key Points:**

- *CT-derived fractional flow reserve (FFRct) is important for monitoring cardiac allograft vasculopathy (CAV) in heart transplant patients.*
- *Over time, transplant patients showed a decrease in distal FFRct and an increase in coronary stenoses.*
- *Temporal changes in FFRct could be crucial for transplant follow-up, aiding in CAV detection.*

## Introduction

Cardiac allograft vasculopathy (CAV) is a major cause of late mortality after heart transplantation (HTx), affecting 1 in 8 heart transplant recipients, as reported by the International Society for Heart and Lung Transplantation (ISHLT) registry [1]. Diagnosing CAV remains challenging, as transplant patients have a denervated heart and rarely present with angina. Consequently, severe CAV in transplant patients often manifests as silent myocardial infarction, allograft function loss, heart failure, or sudden death [2].

The ISHLT recommends annual or biannual invasive coronary angiography (ICA) to assess the development of CAV [3]. However, detecting CAV with ICA is challenging due to the diffuse nature and coronary remodelling of this condition, which limits the sensitivity of ICA [4]. A more sensitive tool is intravascular ultrasonography (IVUS), but IVUS is physically restricted to the larger epicardial arteries and thus cannot evaluate the entire coronary tree [5]. Moreover, ICA and IVUS are invasive modalities with procedural risks [6].

The use of non-invasive modalities has gained prominence in the detection of CAV. Among these modalities, dobutamine stress echocardiography has been utilised; however, its sensitivity is limited [7, 8]. Additionally, rest and stress positron emission tomography (PET) allows quantification of myocardial flow reserve, enabling the assessment of both macro- and microvascular function, which can be valuable in the assessment of CAV [9]. However, it is important to acknowledge that PET has inherent limitations in terms of its high cost and accessibility [9]. Qualitative perfusion cardiac magnetic resonance has limited sensitivity and moderate specificity for detecting CAV, making it less suitable as a screening tool [10, 11]. Quantitative assessments may improve the ability of magnetic resonance imaging to detect CAV [12].

An alternative non-invasive test for routine detection of CAV in HTx patients is Coronary CT angiography (CCTA). CCTA provides imaging of both the coronary lumen and vessel wall at a low radiation dose [13, 14]. A meta-analysis by Wever-Pinzon et al demonstrated excellent sensitivity and negative predictive value for the diagnosis of CAV, being 94 and 99% for the detection of significant CAV and 97% for the presence of any CAV, suggesting it to be a robust technique for excluding CAV [14]. Moreover, CCTA allows non-invasive functional coronary artery evaluation through CT-derived fractional flow reserve (FFRct), which represents the ratio of maximal coronary blood flow through a stenotic artery to the blood flow in the hypothetical case that the artery is normal [15].

We have previously reported on the use of FFRct in HTx patients [16]. In that study, 25% of the HTx patients had focal coronary stenosis with an FFRct value ≤ 0.80 and even without focal stenoses, FFRct values were often abnormal. Changes in FFRct values could serve as an early marker for CAV progression. To our best knowledge, no studies have evaluated the change of FFRct values over time in post-HTx patients. Our study aims to evaluate the change in FFRct values in each of the three major coronary arteries in post-HTx patients over a 2-year period.

## Materials and methods

### Study design and patient selection

We conducted a two-centre retrospective cohort study including HTx patients who underwent two CCTAs approximately 2 years apart, at the Erasmus MC, Rotterdam, the Netherlands and Mount Sinai Hospital, New York, USA. At Erasmus MC, patients routinely undergo annual CCTA assessment starting from the 5th year post-transplant for CAV detection. At Mount Sinai Hospital, a CCTA is performed in HTx patients at the discretion of the treating physicians. At the start of our inclusion, both centres were monitoring 533 transplanted patients in total: 216 patients at the Erasmus MC and 317 patients at Mount Sinai.

**Fig. 1:**
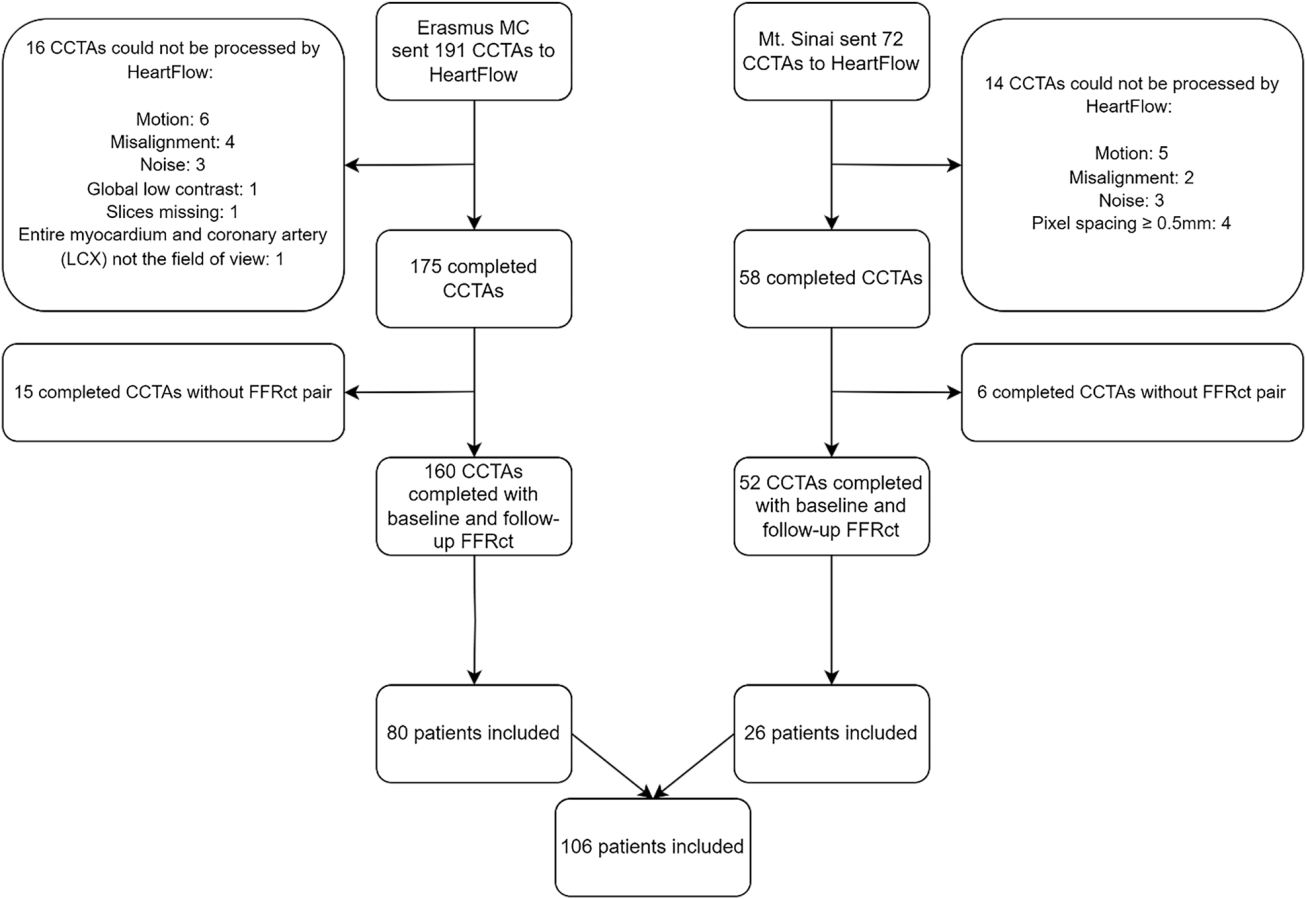
Flowchart of patient inclusion. An overview of the included patients and the reasons why certain cases could not be analysed by HeartFlow Inc. CCTA, coronary computed tomography angiography; FFRct, CT-derived fractional flow reserve

Overall, we aimed to enrol at least 100 patients with a completed FFRct analysis at both baseline and follow-up. Patients were excluded if the scans could not be processed for FFRct analyses (Fig. 1). Our study included patients with coronary stents (*n* = 6) (see below). Notably, FFRct values were limited to coronary arteries without stents. Therefore, our analysis exclusively addressed non-stented arteries. HeartFlow is unable to process cases with two or more systems with metallic stents present (e.g., left circumflex artery (LCX) and left anterior descending artery (LAD)), metallic stents in the presence of left main disease, metallic stents specifically in the left main coronary artery. For each patient, we recorded (at time of baseline CCTA (CCTA_base_)) the patient demographics, angina symptom status, cardiovascular risk factors (diabetes at time of transplant, diabetes at time of CCTA_base_, hypertension, smoking, current medication use, lipid profile (at time of HTx and at time of both CCTAs), donor demographics, coronary calcium scores (for both CCTAs), and coronary events that occurred between both scans (coronary revascularisation and/or myocardial infarction (spontaneous and peri-procedural)). All patients provided written informed consent for the use of their data and FFRct analysis of the CCTA datasets. The study was approved by the Medical Ethical Review Committee (MEC-2017-421) at Erasmus MC and the local Institutional Review Board (20-01526) at Mount Sinai Hospital. This study was conducted in accordance with the ISHLT Ethics statement.

### CCTA and FFRct analysis

A non-contrast-enhanced and contrast-enhanced CCTA examination was performed according to the normal clinical routine on a 256-slice CT scanner (Brilliance iCT, Philips Healthcare), dual-source CT scanner (Force, Siemens Healthineers) or on a Photon-counting CT scanner (NAEOTOM Alpha, Siemens Healthineers). A detailed overview of the CCTA examination parameters is provided in Table 1. The coronary calcium score was calculated on the non-contrast-enhanced scan using the commercially available semi-automatic calcium scoring software (SyngoVia, Siemens). HeartFlow provided the stenosis quantification by using the artificial intelligence-based coronary stenosis quantification (AI-CSQ) software (RoadMap; HeartFlow) [17, 18]. The percent stenosis is automatically computed from the lumen that is segmented from the CCTA, and an idealised or healthy lumen that mimics if stenoses were not present. The percent stenosis calculation is then: 100 * (1 − (patient radius/healthy radius) and then bucketed into CAD-RADS-like categories. Beta-blockers or nitroglycerin were not administered systematically.

**Table 1:** Coronary CT angiography parameters

	CCTA_base_			
Parameter	SOMATOM Drive, Siemens Healthineers	SOMATOM Force, Siemens Healthineers	NAEOTOM Alpha, Siemens Healthineers	Brilliance iCT, Philips Healthcare
Number of patients scanned	19	61	0	26
Heart rate, bpm	71 ± 10	74 ± 10	-	76 ± 10
ECG synchronisation	Prospective	Prospective		Prospective (*n* = 9), Retrospective, (*n* = 17)
kVp (kV)	80, 90, 100, 120	70, 80, 90, 100, 120	-	80, 100, 120
Contrast agent	Ultravist-370	Ultravist-370	-	Isovue-370
CTDI vol (mGy)	9 [7–13]	6 [5–9]	-	23 [16–33]
DLP (mGy·cm)	117 [94–160]	87 [63–115]	-	414 [195–629]
Effective Dose (mSv)	1.6 [1.3–2.2]	1.2 [0.9–1.6]		5.8 [2.7–8.8]

	CCTA_follow-up_			
Number of patients scanned	21	51	8	26
Heart rate, bpm	79 ± 13	78 ± 11	80 ± 11	77 ± 10
ECG synchronisation	Prospective	Prospective	Prospective	Prospective (*n* = 6), Retrospective, (*n* = 20)
kVp (kV)	80, 90, 100	70, 80, 120	120	100, 120
Contrast agent	Ultravist-370, Visipaque	Ultravist-370, Visipaque, Iomeron	Ultravist-370	Isovue-370
CTDI vol (mGy)	7 [6–11]	7 [5–8]	15 [11–16]	33 [22–39]
DLP (mGy·cm)	85 [68–127]	77 [62–100]	200 [158–218]	597 [472–695]
Effective Dose (mSv)	1.8 [1.0–1.8]	1.1 [0.9–1.4]	2.8 [2.2–3.1]	8.4 [6.7–9.7]

*BPM* beats per minute, *CTDIvol* computed tomography dose index volume, *DLP* dose length product, *ECG* electrocardiogram, *kVp* kilovoltage peak, *mSv* millisievert

**Fig. 2:**
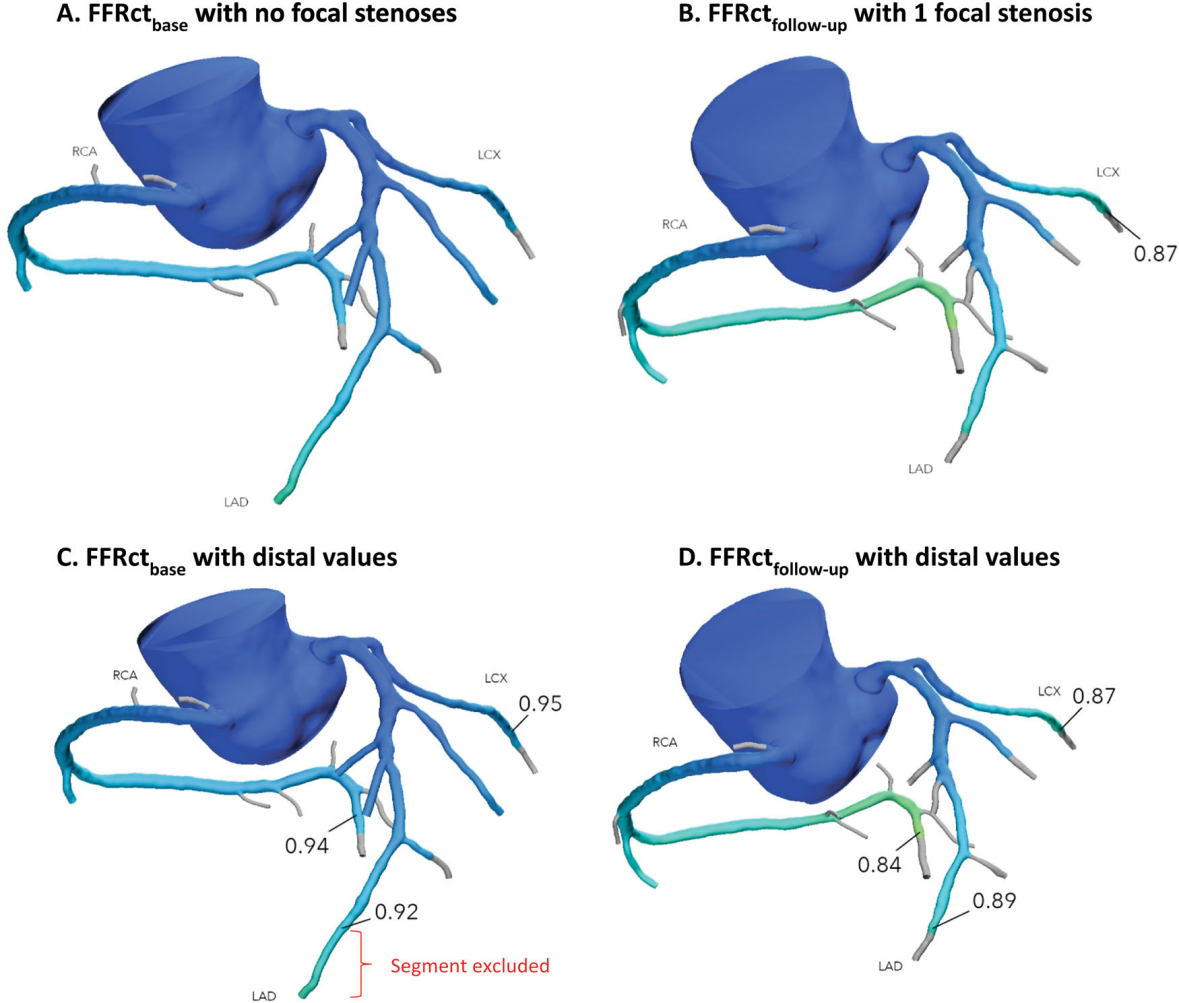
Measurement of the number of focal stenoses and distal CT-derived fractional flow reserve values in a single patient at baseline and follow-up. The focal stenoses (indicated by HeartFlow, Inc.) and distal FFRct values were recorded from the FFRct analyses. **A** shows that the FFRct_base_ has no focal stenosis, whereas the same patient in **B** has one focal stenosis on the FFRct_follow-up_ with a value of 0.87 in the left circumflex artery. **C** displays the FFRct_base_ values at the most distal point of each of the three major coronary arteries, with the investigator placing the measurement pins. Since the length of the segmented coronary arteries varies between FFRct_base_ and FFRct_follow-up_, the shortest segmentation available was used for the measurements. **C** The section distal to the 0.92 measurement point in the left anterior descending artery was disregarded. **D** shows the FFRct_follow-up_ values measured at the same location as in **C**, allowing for a direct comparison between the two analyses. Figure 1S illustrates the multiplanar and volume-rendered images of the distal left circumflex artery stenosis at baseline and follow-up, corresponding to the patient in Fig. 2. FFRct, CT-derived fractional flow reserve

The FFRct analysis was carried out for the CCTA_base_ and the follow-up CCTA (CCTA_follow-up_) for each patient by HeartFlow, Inc., using the same software version (FFR_CT__3.14.0.7). The analysis provides all modelled focal anatomical stenoses of > 30% with their FFRct value measured 2 cm downstream of this stenosis (Fig. 2). FFRct values were measured at the most distal point in each of the three major coronary arteries (right coronary artery (RCA), LAD, and LCX). The length of the segmented part of the coronary artery sometimes varied between both analyses. Therefore, both FFRct analyses for each patient were placed side by side, ensuring that the measurement of the most distal point was at exactly the same location for both scans using the interactive viewer (Fig. 2).

To obtain the overall distal FFRct value per patient, we calculated the mean of the distal FFRct values across all three coronary arteries. We included the number of modelled > 30% focal stenoses and the overall distal FFRct value in the analysis only for vessels with available FFRct values in both scans. Subgroup analysis was performed by stratifying patients according to delta FFRct, using a cut-off value of 0.06. The first group consisted of patients with an FFRct drop of less than 0.06, while the second group included patients with an FFRct drop equal to or more than 0.06. This cut-off value was chosen based on the limits of agreement reported for FFRct in a previous study on its reproducibility, which demonstrated an upper limit of agreement of 0.08 and a lower limit of agreement of 0.06 for FFRct [19]. An FFRct value ≤ 0.80 is considered to indicate a hemodynamically significant stenosis [20].

### Statistical analysis

Continuous variables are expressed as mean ± standard deviation or median [interquartile range] depending on the distribution. Categorical variables are expressed as frequencies with percentages. Patient subgroups were compared by unpaired *t*-test, Wilcoxon signed-rank test, Kruskal–Wallis test, Mann–Whitney *U*-test, chi-square or Fisher’s exact test depending on the type of data. Proportions of patients with hemodynamically significant focal stenoses between baseline and follow-up were compared by, McNemar’s test. Normality was tested by the Shapiro-Wilk test. A two-tailed *p*-value of < 0.05 was considered statistically significant. All statistical analyses were performed using SPSS statistical software (IBM Corp. IBM SPSS Statistics for Windows, Version 28.0.1.0 Armonk, NY: IBM Corp).

## Results

### Patient characteristics

After the exclusion of scans that could not be processed for FFRct, a total of 106 HTx patients were included with paired CCTA and FFRct analysis: 71 Males (67%), aged at the time of CCTA_base_ 57 [47–67] years, 9 [6–13] years after HTx. None of the patients had angina symptoms or reduced left ventricular ejection fraction. Non-ischaemic cardiomyopathy was the most prevalent primary diagnosis for HTx (62%). One-hundred and five patients (99%) were on calcineurin inhibitors, 54 (51%) on steroids, 42 (40%) on mycophenolate, 18 (17%) patients on mammalian target of rapamycin inhibitors and 1 patient (1%) were on purine antagonists. Ninety-one patients (87%) used statins and/or ezetimibe at the time of CCTA_base_ (Table 2).

**Table 2:** Baseline patient characteristics

		Total	FFRct drop < 0.06	FFRct drop ≥ 0.06	*p-*value
Total number of patients, *n*		106	86	20	
Age, years		57 [47–67]	57 [48–67]	53 [30–65]	0.25
Recipient gender, % male		71 (67%)	60 (70%)	11 (55%)	0.21
Race	White	88 (83%)	72 (84%)	16 (80%)	0.19
	Black or African American	4 (4%)	2 (2%)	2 (10%)	
	Asian	4 (4%)	2 (2%)	2 (10%)	
	Turkish	1 (1%)	1 (1%)	0 (0%)	
	Moroccan	1 (1%)	1 (1%)	0 (0%)	
	Other	8 (8%)	8 (9%)	0	
Ethnicity	Non-Hispanic or Latino	101 (95%)	81 (94%)	20 (100%)	0.58
	Hispanic or Latino	5 (5%)	5 (6%)	0 (0%)	
Body-mass index, kg/m^2^		26 ± 5	25 ± 5	27 ± 5	0.24
Angina status	Typical	0 (0%)	0 (0%)	0 (0%)	-
	Atypical	0 (0%)	0 (0%)	0 (0%)	
	None	106 (100%)	86 (100%)	20 (100%)	
Diabetes mellitus	Prior to HTx^a^	6 (6%)	4 (5%)	2 (11%)	0.60
	At the time of CCTA_base_	28 (26%)	23 (27%)	5 (25%)	0.87
Insulin use	At the time of HTX^b^	3 (3%)	2 (3%)	1 (5%)	0.50
	At the time of CCTA_base_	12 (11%)	10 (12%)	2 (10%)	1.00
	At the time of CCTA_follow-up_	12 (11%)	10 (12%)	2 (10%)	1.00
Hypertension		81 (76%)	68 (79%)	13 (65%)	0.18
Smoking	Current	3 (3%)	3 (4%)	0 (0%)	0.63
	Past	19 (18%)	16 (19%)	3 (15%)	
	Never	84 (79%)	67 (78%)	17 (85%)	
Left ventricular ejection fraction < 50%		0 (0%)	0 (0%)	0 (0%)	-
Time since HTx at baseline CCTA, years		9 [6–13]	8 [6–13]	9 [7–13]	0.40
Primary diagnosis for HTx	Cardiomyopathy	66 (62%)	52 (61%)	14 (70%)	0.57
	Ischaemic heart disease	32 (30%)	28 (33%)	4 (20%)	
	Valvular heart disease	4 (4%)	3 (4%)	1 (5%)	
	Congenital heart disease	2 (2%)	1 (1%)	1 (5%)	
	Re-transplant	2 (2%)	2 (2%)	0 (0%)	
Recipient age at HTx, years		49 [36–56]	49 [38–57]	45 [14–56]	0.29
Donor age, years		45 [25–52)	46 [25–52)	40 [14–55)	0.63
Donor gender, % male		41 (47%)	33 (49%)	8 (42%)	0.62
Donor body-mass index, kg/m^2^		23 ± 4	23 ± 3	22 ± 5	0.23
CMV within the first year post-HTx		15 (14%)	12 (14%)	3 (15%)	1.00
Number of cellular-mediated rejection		51 (48%)	40 (47%)	11 (55%)	0.49
Number of antibody-mediated rejection		2 (2%)	2 (2%)	0 (0%)	1.00
Patients with coronary stent in cardiac transplant		6 (8%)	4 (7%)	2 (11%)	0.62
Pacemaker present in cardiac transplant		19 (18%)	14 (16%)	5 (25%)	0.36
Statin and/or Ezetimibe use	At the time of CCTA_base_	91 (87%)	74 (87%)	17 (85%)	0.73
	At the time of CCTA_follow-up_	96 (91%)	76 (88%)	20 (100%)	0.20
Thrombocyte aggregation inhibitors and/or oral anticoagulant use	At the time of CCTAbase	100 (95%)	80 (94%)	20 (100%)	0.58
	At the time of CCTA_follow-up_	100 (94%)	80 (93%)	20 (100%)	0.59
Current immunosuppressive regimen	Mammalian target of rapamycin inhibitors	18 (17%)	12 (14%)	6 (30%)	0.085
	Calcineurin inhibitor	105 (99%)	85 (99%)	20 (100%)	1.00
	Steroids	54 (51%)	46 (54%)	8 (40%)	0.28
	Mycophenolate	42 (40%)	35 (41%)	7 (35%)	0.64
	Purine antagonists	1 (1%)	1 (1%)	0 (0%)	1.00
Serum creatinine, umol/L	At the time of CCTA_base_	105 ± 24	105 ± 24	106 ± 27	0.85
	At the time of CCTA_follow-up_	108 ± 43	108 ± 47	107 ± 24	0.95
Cholesterol, mmol/L	At the time of CCTA_base_	4.4 ± 1.0	4.4 ± 1.1	4.2 ± 0.9	0.33
	At the time of CCTA_follow-up_	4.3 ± 0.9	4.4 ± 0.9	4.1 ± 0.8	0.26
Triglycerides, mmol/L	At time of CCTA_base_	1.5 [1.0–2.1]	1.5 [1.0–2.1]	1.4 [1.1–1.7]	0.38
	At the time of CCTA_follow-up_	1.4 [1.1–1.9]	1.5 [1.2–2.0]	1.2 [0.9–1.8]	0.13
HDL, mmol/L	At the time of CCTA_base_	1.5 [1.1–1.7]	1.5 [1.1–1.7]	1.6 [1.1–1.7]	0.47
	At the time of CCTA_follow-up_	1.5 ± 0.4	1.5 ± 0.4	1.5 ± 0.4	1.00
LDL, mmol/L	At the time of CCTA_base_	2.6 ± 0.9	2.6 ± 0.9	2.5 ± 0.7	0.41
	At the time of CCTA_follow-up_	2.4 ± 0.7	2.4 ± 0.7	2.3 ± 0.6	0.61
CCS at the time of CCTA_base_, Agatston units	Total	0 [0–37]	0 [0–31]	0 [0–98]	0.72
	RCA	0 [0–2]	0 [0–2]	0 [0–3]	0.79
	LAD	0 [0–23]	0 [0–19]	0 [0–75]	0.62
	LCX	0 [0–0]	0 [0–0]	0 [0–6]	0.54
CCS at the time of CCTA_follow-up,_ Agatston units	Total	3 [0–85]	3 [0–67]	1 [0–151]	0.75
	RCA	0 [0–13]	0 [0–14]	0 [0–11]	1.00
	LAD	0 [0–42]	0 [0–40]	0 [0–118]	0.66
	LCX	0 [0–2]	0 [0–1]	0 [0–9]	0.79

Data is presented as mean ± standard deviation, median [25th–75th percentile] or frequencies (percentage)

*CAV* cardiac allograft vasculopathy, *CCS* coronary calcium score, *CCTA* coronary computed tomography angiography, *CCTAbase* baseline CCTA, *CCTAfollow-up* follow-up CCTA, *CMV* cytomegalovirus, *HDL* high-density-lipoprotein, *HTx* heart transplantation, *LAD* left anterior descending artery, *LCX* left circumflex artery, *LDL* low-density lipoprotein, *RCA* right coronary artery

^a^ Available for 94 patients

^b^ Available for 93 patients

The median time between baseline and follow-up CCTA was 23 [22–24] months and coronary calcium score increased from 0 [0–37] at baseline to 3 [0–85] at 2-year follow-up (*p* < 0.001). Significant stenosis (≥ 50%) on CCTA increased from baseline to follow-up. Initially, 26 patients (25%) presented with significant stenosis in any vessel at CCTA_base_, which increased to 52 patients (49%) at CCTA_follow-up_ (*p* < 0.001). Detailed vessel-specific CCTA results are presented in Table 3.

**Table 3:** Coronary computed tomography angiography and CT-derived fractional flow reserve results

		CCTA_base_	CCTA_follow-up_	*p-*value
Number of patients with significant stenosis (≥ 50%) on CCTA in any vessel		26 (25%)	52 (49%)	< 0.001
Number of patients with significant stenosis (≥ 50%) on CCTA per vessel	RCA	15 (14%)	23 (22%)	0.134
	LAD	11 (10%)	26 (25%)	0.009
	LCX	9 (9%)	20 (19%)	0.035

		FFRct_base_	FFRct_follow-up_	
Overall distal FFRct		0.89 [0.86–0.92]	0.87 [0.84–0.91]	< 0.001
Distal FFRct per vessel	RCA	0.92 [0.86–0.95]	0.90 [0.86–0.94]	0.004
	LAD	0.85 [0.79–.0.90]	0.84 [0.76–0.90]	0.001
	LCX	0.92 [0.88–0.96]	0.91 [0.85–0.95]	0.009
Number of patients with overall distal FFRct ≤ 0.80		5 (5%)	17 (16%)	0.003
Number of patients with distal FFRct ≤ 0.80	RCA	6 (6%)	13 (12%)	0.12
	LAD	29 (27%)	37 (35%)	0.13
	LCX	7 (7%)	10 (9%)	0.58
Overall number of modelled focal stenoses > 30% on the FFRct analysis		1 (0–2)	2 (0–3)	0.009
Number of patients with at least one hemodynamically significant modelled focal stenosis > 30% on the FFRct analysis (FFRct ≤ 0.80) in any vessel		31 (29%)	44 (42%)	0.042
Number of patients with at least one hemodynamically significant modelled focal stenosis > 30% on the FFRct analysis (FFRct ≤ 0.80) per vessel	RCA	9 (8%)	10 (9%)	0.80
	LAD	19 (18%)	32 (30%)	0.024
	LCX	10 (9%)	13 (12%)	0.47

Overall distal FFRct is the mean of the three distal FFRct values. Overall FFRct of focal stenosis is the mean of the data presented as mean ± standard deviation (SD), median [25th–75th percentile], or frequencies (percentage)

*FFRct* CT-derived fractional flow reserve, *LAD* left anterior descending artery, *LCX* left circumflex artery, *RCA* right coronary artery

Between CCTA_base_ and CCTA_follow-up_ 11 (10%) patients underwent ICA. None of the patients underwent revascularisation. None of the patients suffered from a procedural or spontaneous myocardial infarction.

### FFRct analysis

**Fig. 3:**
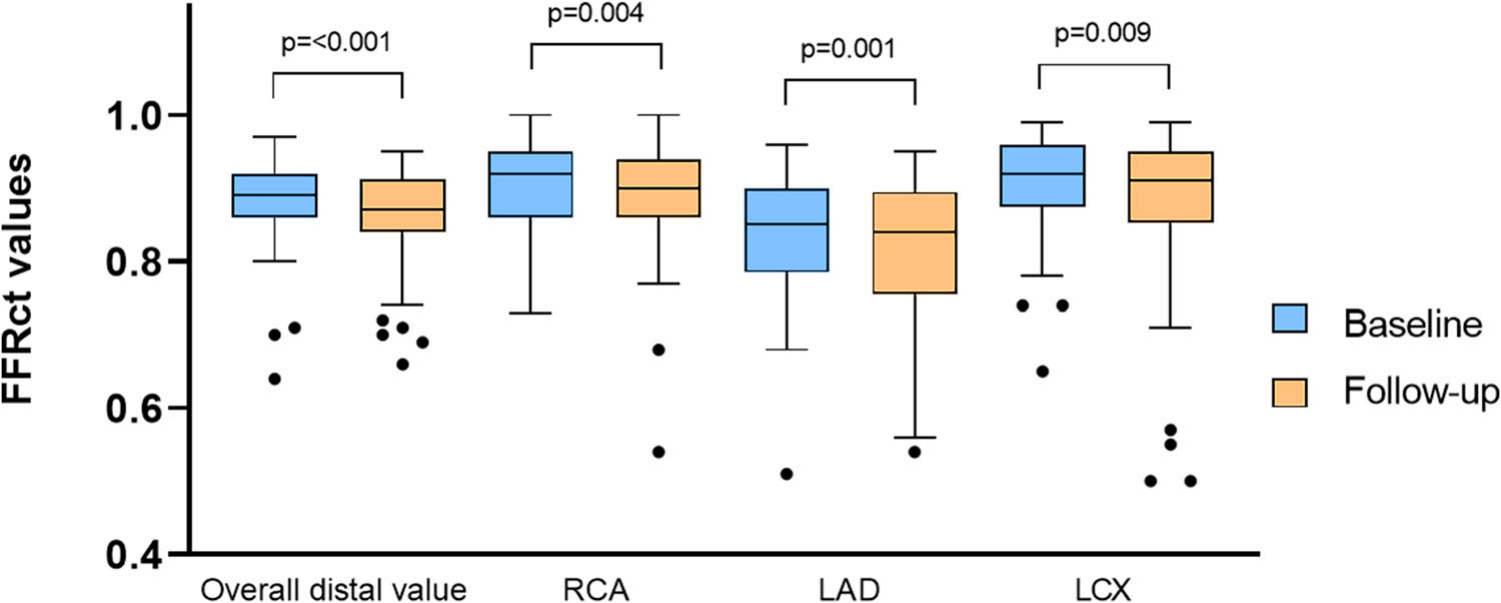
Boxplots comparing FFRct values between baseline and follow-up. Box plot showing the distribution of the overall and per-vessel distal FFRct values for the baseline (blue) and follow-up (orange) measurements. The boxes represent the interquartile range (IQR), with the horizontal line inside indicating the median value. The whiskers extend to the minimum and maximum values within 1.5 times the IQR (interquartile range) below the first quartile and above the third quartile. FFRct, CT-derived fractional flow reserve; LAD, left anterior descending artery; LCX, left circumflex artery; RCA, right coronary artery

FFRct analyses were carried out in all patients for both scans. Initially, 263 CCTA’s were submitted for analysis, of which 30 cases could not be processed (Flowchart Fig. 1). Of the potential coronary arteries available for analysis (*n* = 318; 106 patients × 3 vessels per patient) 311 (98%) had an FFRct analysis available at baseline and 309 (97%) at follow-up. Overall, 309 coronary arteries had an analysis at both baseline and follow-up. In one FFRct analysis, the value for the LCX could not be calculated due to a chronic total occlusion. FFRct analyses could not calculate the LCX or LAD value due to the presence of a coronary stent in 2 and 10 cases, respectively. In three FFRct analyses, the value of the RCA could not be calculated due to substantial CCTA artefacts. The overall distal FFRct value was higher for FFRct_base_ compared to FFRct_follow-up_. The median distal FFRct values significantly decreased in all three vessels from baseline to follow-up. (Table 3 and Fig. 3). The median delta FFRct between the distal FFRct_base_ value and the distal FFRct_follow-up_ value was not significantly different for all three vessels (RCA: −0.01 [−0.04 to 0.01]); LAD: −0.02 [−0.06 to 0.02]; LCX −0.01 [−0.03 to 0.01]; *p* = 0.95.

For the comparison of the focal stenosis in both scans, we excluded the coronary arteries that did not have an FFRct analysis in both scans. Focal stenoses were more prevalent in the FFRct_follow-up_ compared to the FFRct_base_ (number of focal stenoses: 1 [0–3] vs. 2 [0–3], *p* = 0.009). The number of patients with at least one hemodynamically significant focal stenosis in any vessel was higher at the time of the FFRct_follow-up_ compared to FFRct_base_ (31 (29%) vs. 44 (42%), *p* = 0.042). The highest proportion of patients with a hemodynamically significant stenosis was observed in the left anterior descending artery (LAD), with 19 (18%) on FFRct_base_ and 32 (30%) on FFRct_follow-up_, indicating an increase over time (*p* = 0.024). The number of patients with hemodynamically significant stenosis in the right coronary artery (RCA) and the left circumflex artery (LCX) was overall lower. Nine (8%) patients had at least one hemodynamically significant focal stenosis in the RCA at baseline and 10 (9%) had a stenosis at follow-up, demonstrating no significant increase (*p* = 0.80). For the LCX, 10 (9%) and 13 (12%) patients had stenosis at baseline and follow-up, respectively, indicating no significant increase (*p* = 0.47). (Table 3).

### Stratification of patients based on delta FFR

Of 106 patients, 86 (81%) patients had a drop of the overall distal FFRct value < 0.06 and 20 (19%) of ≥ 0.06. The baseline demographic and clinical characteristics of the two study groups were comparable (Table 2). The subgroup analysis identified no association between baseline FFRct variables and a larger FFRct drop ( ≥ 0.06). The presence of significant stenosis on CCTA was also not associated with a larger FFRct drop. (Table 1S).

### Stratification of vessels based on stenoses development

In vessels that developed new significant stenoses from baseline to follow-up, the distal FFRct significantly decreased from 0.89 [0.84–0.94] at baseline to 0.86 [0.70–0.92] at follow-up (*p* < 0.001). In vessels without the development of significant stenoses from baseline to follow-up, the distal FFRct also decreased significantly from 0.90 [0.85–0.94] at baseline to 0.89 [0.84–0.94] at follow-up (*p* = 0.003).

## Discussion

Our study describes the first cohort of post-heart transplant patients with two FFRct analyses performed with a median of 2 years apart, providing valuable insight into functional changes of the coronary arteries after HTx. Our study findings revealed an increase in the number of focal coronary stenoses and a decrease in distal FFRct values in all three major coronary arteries between baseline and follow-up assessments. This raises the question of whether early intervention could affect the progression of CAV.

Since CAV is described as a diffuse and concentric narrowing of large epicardial and small intramyocardial arteries, not only anatomical assessment but also the functional evaluation of the coronary arteries is important. Dobutamine stress echocardiography has traditionally been the most common non-invasive imaging modality used for the detection of CAV, but it has modest specificity and, particularly, sensitivity. Nonetheless, a negative stress echocardiogram has a high negative predictive value [21]. Similarly, single-photon emission computed tomography has prognostic value but limited diagnostic accuracy [21]. PET offers higher spatial resolution and, importantly, can provide quantitative measurements of myocardial flow reserve, which, when reduced, predict an increased risk of adverse events [21, 22].

Regarding catheter-based functional evaluation of the coronary arteries, invasive FFR can be useful in predicting adverse clinical outcomes in post-HTX patients [23, 24]. In CAV, invasive FFR correlates with IVUS-assessed plaque volume and an FFR value of ≤ 0.80 has been observed in 15% of asymptomatic post-HTX patients with normal ICAs [24]. While not specifically tested in HTx patients, FFRct in stable chest pain patients has a good correlation and reproducibility of measurements with invasive FFR as the reference standard [15, 25, 26]. FFRct could therefore play an essential role in the follow-up of post-HTx patients by adding functional information to CCTA, which has an established role in excluding CAV. Considering the ISHLT recommendation for (bi)annual ICA for CAV evaluation, the integration of FFRct into follow-up assessments may lead to a decrease in unnecessary ICAs [3]. Key advantages of FFRct include its non-invasive approach using already acquired CCTA data and that it can calculate FFR values for each point in all coronary arteries giving detailed functional information of the complete coronary tree.

Our findings show a significant decrease in the distal FFRct values of all three coronary arteries over the 2-year follow-up period. A likely explanation for this reduction is the progression in the anatomic burden of CAV, as suggested by the correlation between invasive FFR and IVUS-based plaque volume. Indeed, we observed an increase in the number of > 30% stenoses; however, it is important to note that while the decrease in FFRct values is more pronounced in vessels that developed new significant stenoses, a reduction is also observed in vessels without stenosis development. While we were unable to quantify vessel wall or luminal volume, it is plausible that other mechanisms may also influence the observed outcomes. These could include changes in endothelial and microvascular function, local stenosis features (such as location, geometry or plaque composition), or left ventricular mass [27, 28], indicating the potential need for early intervention to improve or delay the onset of CAV. Nonetheless, for 84% of the patients, the average distal FFRct value at the time of follow-up remained > 0.80, a threshold associated with a low risk of adverse events such as death or myocardial infarction in stable chest pain patients [29].

In addition to the distal FFRct value, the degree of reduction in FFRct value may offer relevant information for the management of post-HTx patients. Patients experiencing a greater decrease in FFRct values may have higher rates of CAV progression compared to those with a smaller reduction in FFRct values. This information may aid in identifying patients at increased risk of adverse outcomes and timely adjusting medical therapy. For patients with FFRct values > 0.80 and a substantial drop in FFRct, optimising medical therapy may be necessary to prevent adverse outcomes, as these patients may not show epicardial disease appropriate for revascularisation, but already have early microvascular dysfunction [21]. In our study, we observed an overall decrease in distal FFRct value of 0.02 over a 2-year period. This finding raises the question of whether patients who experience a larger decrease in FFRct, exceeding 0.02 over the same period, may have worse clinical outcomes. Future studies investigating this relationship could provide valuable insights into the clinical utility of serial FFRct measurements in heart transplant recipients.

In a recent study by Ahn et al, the diagnostic performance of combined CCTA and CT-myocardial perfusion imaging for CAV was explored, suggesting it as a potent non-invasive screening method for early detection [30]. However, their findings revealed limited diagnostic accuracy of CT-FFR in detecting CAV in post-heart transplant patients. Ahn et al attributed this limitation to the progression of microvascular dysfunction over time after transplantation (≥ 2 years), where (invasive) FFR can no longer accurately represent the microvasculature status [31]. In contrast, our study had a median time of 10 years between transplantation and baseline CT, differing from Ahn et al’s 39-month duration. Therefore, the findings of Ahn et al may not be directly applicable to our population of post-heart transplant patients with a longer post-transplantation period.

A pilot study conducted by Oebel et al has further highlighted the significance of CT-perfusion imaging in CAV [32]. This study showed that a combined CCTA and CT-perfusion imaging protocol could serve as a ‘one-stop-shop’ approach enabling both morphological and functional assessment in post-heart transplant patients. Patients with hemodynamically significant CAV were correctly identified with this approach. These findings, combined with the high diagnostic accuracy and safety for detecting CAV, endorse CCTA as an effective alternative for ICA in the follow-up of HTx patients, also offering functional insights through FFRct or CT-perfusion imaging [13, 14].

There are some limitations in this study. Firstly, we have selected patients with two consecutive CCTAs to analyse the FFRct values over time. This approach introduces a potential selection bias, as patients without follow-up CCTAs might have experienced adverse events. Secondly, it is important to acknowledge that six cases were excluded from the analyses due to the presence of stents in two or more coronary systems. This might also introduce a selection bias, favouring the inclusion of patients with less advanced transplant vasculopathy in this heart transplant population. These potential biases could explain, at least in part, the absence of observed adverse events in our study population. The exclusion of these cases was driven by the limitations of the current HeartFlow application, which does not support analyses in cases involving multiple stents. However, this subset of patients accounts for only a small proportion of our total study population, and therefore, the extent to which we are underestimating the disease burden and progression remains uncertain. Additionally, it is important to note that our study did not employ a reference standard method, such as ICA, for validating the FFRct and CCTA findings. Consequently, analyses of sensitivity and specificity comparing FFRct with CCTA were not conducted. Furthermore, our study also does not include HTx patients with poor kidney function or contrast allergy, as these patients did not undergo CCTA. Additionally, the return rate of the HeartFlow FFRct analyses in our study was 11%. This return rate is inherent to the study population. HTX patients generally have higher heart rates which may impact the quality of images and influence the return rate. Although this is the first study with follow-up FFRct analyses, a follow-up time of 2 years between both analyses is relatively limited. The observed overall decrease in the distal FFRct value of 0.02 over the 2-year period is minimal. However, this modest decline can be attributed to the short time interval between the scans. With a more extended follow-up period, it is likely that a greater decrease would be observed. Given that the median time post-transplant duration for patients in our study was 9 years, our results may not adequately reflect the early post-transplant period, which can also be relevant for identifying significant changes in FFRct values. Additionally, longer follow-up is necessary to investigate the prognostic value of FFRct in post-HTX patients.

In conclusion, our findings underscore the integral role of CCTA alongside FFRct in monitoring CAV. We observed a decrease in the distal coronary FFRct values in post-HTX patients in each of the three major coronary arteries and an increase in the number of focal coronary stenoses over 2 years. The temporal FFRct change rate may become an additional parameter in the follow-up of HTx patients, but more research is needed to elucidate its role.

## Supplementary information

ELECTRONIC SUPPLEMENTARY MATERIAL

## Abbreviations

CAV: Cardiac allograft vasculopathy
CCTA: Coronary computed tomography angiography
CCTA_base_: Baseline CCTA
CCTA_follow-up_: Follow-up CCTA
FFR: Fractional flow reserve
FFRct: CT-derived fractional flow reserve
HTx: Heart transplantation
ICA: Invasive coronary angiography
ISHLT: International Society for Heart and Lung Transplantation
IVUS: Intravascular ultrasound
LAD: Left anterior descending artery
LCX: Left circumflex artery
PET: Positron emission tomography
RCA: Right coronary artery
