# Supplementary material for: Temporal changes in CT-derived fractional flow reserve in patients after heart transplantation
Source: Eur Radiol. 2024 Jul 17;35(1):232–43. doi: 10.1007/s00330-024-10932-z (PMC11631993; doi:10.1007/s00330-024-10932-z)
Supplement: Supplementary file 1 — ELECTRONIC SUPPLEMENTARY MATERIAL [file 330_2024_10932_MOESM1_ESM.pdf]

Temporal changes in CT-derived fractional flow reserve in patients after heart transplantation

ELECTRONIC SUPPLEMENTARY MATERIAL

Table 1S. Subgroup Analysis of CCTA and Baseline FFRct Results Based on FFRct Drop (≥0.06)

		**FFRct drop <0.06 (n=86)**	**FFRct drop ≥0.06 (n=20)**	**P value**
Number of patients with significant stenosis (≥50%) on CCTA in any vessel at baseline		21 (24%)	5 (25%)	0.41
Number of patients with significant stenosis (≥50%) on CCTA per vessel at baseline	RC A	14 (16%)	1 (5%)	0.49
	LAD	8 (9%)	3 (15%)	0.11
	LCX	7 (8%)	2 (10%)	0.043
Overall distal FFRct baseline		0.88 [0.86-0.92]	0.89 [0.87-0.93]	0.43
Distal FFRct per vessel baseline	RCA	0.92 [0.86-0.95]	0.94 [0.88-0.95]	0.38
	LAD	0.86 [0.78-0.90]	0.85 [0.79-0.89]	0.72
	LCX	0.92 [0.87-0.96]	0.93 [0.89-0.95]	0.66
Number of patients with overall distal FFRct ≤0.80 at baseline		4 (5%)	1 (5%)	1.00
Number of patients with distal FFRct ≤0.80 at baseline	RCA	6 (7%)	0 (0%)	0.59
	LAD	24 (28%)	5 (25%)	1.00
	LCX	6 (7%)	1 (5%)	1.00
Overall number of focal stenoses		1 [0-3]	1 [0-2]	0.24
Number of patients with at least one hemodynamically significant focal stenosis (FFRct ≤ 0.80) in any vessel at baseline		28 (33%)	3 (15%)	0.17
Number of patients with at least one hemodynamically significant focal stenosis (FFRct ≤ 0.80) per vessel at baseline	RCA	9 (11%)	0 (0%)	0.20
	LAD	18 (21%)	1 (5%)	0.12
	LCX	8 (9%)	2 (10%)	1.00
Overall distal FFRct is the mean of the three distal FFRct values. Overall FFRct of focal stenosis is the mean of the Data is presented as mean ± standard deviation (SD), median [25th – 75th percentile], or frequencies (percentage). FFRct = CT-derived Fractional Flow Reserve; LAD = Left Anterior Descending Artery; LCX = Left Circumflex Artery; RCA: Right Coronary Artery.				

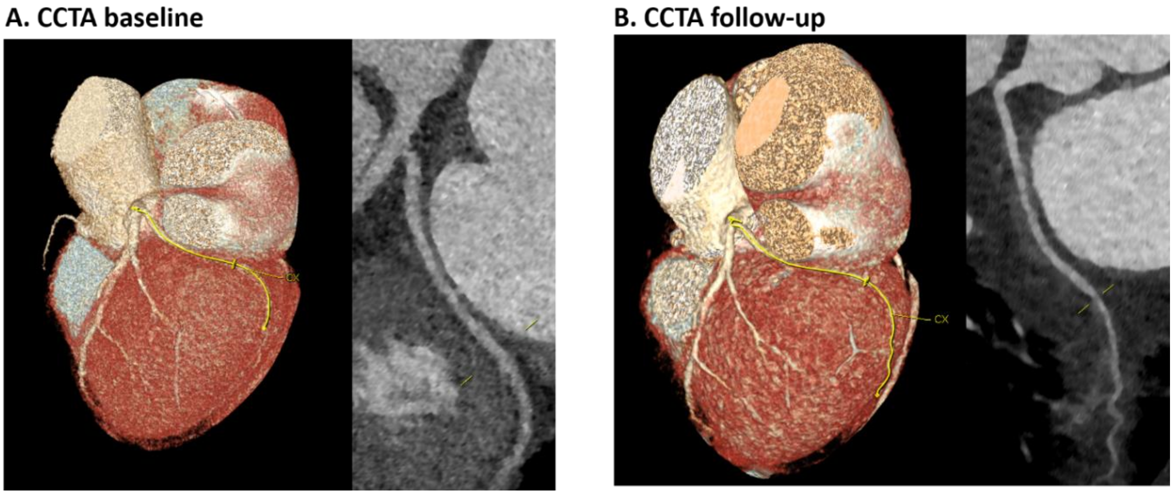

Figure 1S. Multiplanar and Volume Rendered Images of Distal Left Circumflex Artery Stenosis at Baseline and Follow-Up, Corresponding to the Patient Presented in Figure 2
